# Developments in Fatty Acid-Derived Insect Pheromone Production Using Engineered Yeasts

**DOI:** 10.3389/fmicb.2021.759975

**Published:** 2021-11-11

**Authors:** Xiaoling Zhang, Qin Miao, Xia Xu, Boyang Ji, Lingbo Qu, Yongjun Wei

**Affiliations:** ^1^Key Laboratory of Advanced Drug Preparation Technologies, Ministry of Education, School of Pharmaceutical Sciences, Zhengzhou University, Zhengzhou, China; ^2^Laboratory of Synthetic Biology, Zhengzhou University, Zhengzhou, China; ^3^Department of Biology and Biological Engineering, Chalmers University of Technology, Gothenburg, Sweden; ^4^College of Chemistry, Zhengzhou University, Zhengzhou, China

**Keywords:** insect sex pheromone, fatty acids, *Saccharomyces cerevisiae*, metabolic engineering, synthetic biology

## Abstract

The use of traditional chemical insecticides for pest control often leads to environmental pollution and a decrease in biodiversity. Recently, insect sex pheromones were applied for sustainable biocontrol of pests in fields, due to their limited adverse impacts on biodiversity and food safety compared to that of other conventional insecticides. However, the structures of insect pheromones are complex, and their chemical synthesis is not commercially feasible. As yeasts have been widely used for fatty acid-derived pheromone production in the past few years, using engineered yeasts may be promising and sustainable for the low-cost production of fatty acid-derived pheromones. The primary fatty acids produced by *Saccharomyces cerevisiae* and other yeasts are C16 and C18, and it is also possible to rewire/reprogram the metabolic flux for other fatty acids or fatty acid derivatives. This review summarizes the fatty acid biosynthetic pathway in *S. cerevisiae* and recent progress in yeast engineering in terms of metabolic engineering and synthetic biology strategies to produce insect pheromones. In the future, insect pheromones produced by yeasts might provide an eco-friendly pest control method in agricultural fields.

## Introduction

Pheromones are the chemicals used by individuals to communicate with members of the same species (Karlson and Luscher, 1959). Many insect pheromones are fatty acid-derived molecules, which play an essential role in the insect life cycle, such as attraction, aggression, aphrodisiacs, anti-aphrodisiacs, aggregation, kin recognition, and alarm signaling (Yew and Chung, 2015). Insect pheromones are trace chemicals usually secreted by female insects to attract males of the same species. Most insect pheromones studies focus on moth and a few other Lepidopteran sex pheromones. Based on their chemical structures, moth sex pheromones are classified into three types, the Type-I (75%), the Type-II (15%), and the miscellaneous groups (10%) (Ando et al., 2004). Type-I sex pheromones are alcohols, aldehydes, and acetates with 10–18 carbon chains, and are produced by most moth species. Type-II sex pheromones comprise odd polyunsaturated hydrocarbons (C17-C23) with two or three double bonds at positions three, six, or nine, in addition to their corresponding epoxide derivatives (Matsumoto, 2010; Sun et al., 2019; Yang et al., 2020). The miscellaneous groups of sex pheromones comprise secondary alcohols with short-chain fatty acids (C7 and C9) and can be further classified as Type-0, while those with methyl-branched compounds are classified as Type-III (Ando and Yamamoto, 2020).

Pest control is a global challenge associated with food supplies. One of the most potent green strategies for pest biocontrol involves mating disruption in insects by releasing pheromones into the crop field (Reddy and Guerrero, 2010; Benelli et al., 2019). Sex pheromones have been widely used for insect control and monitoring in agriculture, horticulture, and forestry (Witzgall et al., 2010). Using pheromones for pest control is highly efficient, non-toxic, not harmful to the beneficial insect species, and does not pollute the environment, which satisfies the requirement of food and environmental security. The sex pheromone components are already being chemically synthesized, nevertheless, their chemical synthesis requires expensive substrates and catalysts and generates hazardous wastes (Herbert et al., 2013; Turczel et al., 2018). In this study, we review Type-I insect sex pheromones and their biosynthetic pathways, since most sex pheromones are assigned to this class. We have also summarized the biosynthesis of sex pheromones using engineered yeasts and discussed the potential application of yeasts for insect sex pheromone production.

## Sex Pheromone Biosynthesis in Lepidopterans

Moths and other Lepidopteran insects mainly produce Type-I sex pheromones; these are produced in the sex pheromone gland (PG), and released when needed (Tillman et al., 1999; Ando et al., 2004). In fact, most female moths release pheromones at relatively low level, while some species release periodically when matched pheromones are available (Raina et al., 2000; Jurenka, 2017).

**FIGURE 1:**
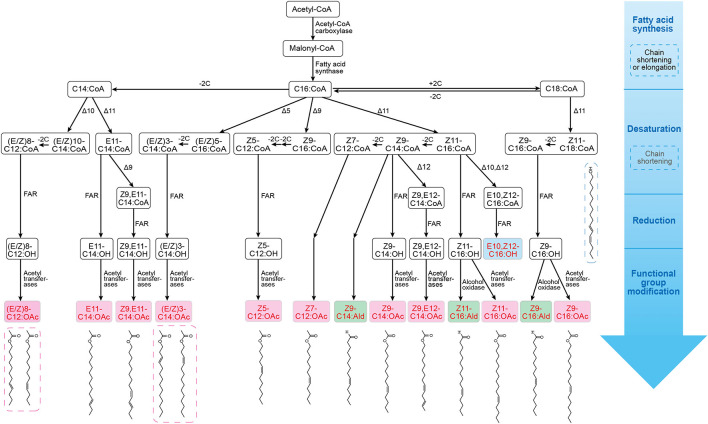
Biosynthetic pathways of sex pheromones in several lepidopteran species (including *Bombyx mori*, *Spodoptera litura*, *Streltzoviella insularis*, *Antheraea pernyi*, *Thysanoplusia intermixta*, *Heliothis subflexa*, *H. virescens*, *Grapholita molesta*, and *G. dimorpha*). Pheromone synthesis starts with a ubiquitous fatty acid synthesis, followed by desaturation of fatty acyl-CoA precursors, then reduction and functional group modification steps to produce species-specific alcohol (blue), aldehyde (green), or acetate ester (pink), that can be used as pheromone blends; the structure of each pheromone molecule is also shown. The general biosynthesis processes and enzymes involved are also marked in this figure.

Since most moth pheromones have a straight-chain carbon backbone, their biosynthetic pathways are common in different moth species (Figure 1). The first step of sex pheromone biosynthesis is the synthesis of fatty acids, which starts with acetyl-CoA and is catalyzed by acetyl-CoA carboxylase and fatty acid synthase (FAS). A series of saturated fatty acid precursors are used for pheromone biosynthesis. The secondary step is desaturation, which involves highly specific desaturases to generate one double bond or several double bonds at different positions in the fatty acids. The third step involves the reduction of fatty acids by fatty acid reductases (FARs) to convert fatty acyl to fatty alcohol. The succeeding steps are associated with the functional group modification catalyzed by fatty alcohol oxidases and fatty acetyltransferases to form aldehyde and acetate ester groups (Tehlivets et al., 2007; Wang M. et al., 2020). Here, we highlight some of the key enzymes involved in the desaturation, reduction, and functional group modification steps in the biosynthesis of representative pheromones (Supplementary Figure 1).

## Desaturases

In Lepidoptera and other insects, a variety of acyl-CoA desaturases catalyze the desaturation of fatty acyl intermediates, and they often localize at endoplasmic reticulum membrane (Figure 1; Hagström et al., 2013a). Especially, Δ11 desaturases (Z11 desaturase, E11 desaturase) generate a double bond at the C11 position. In the female silkmoth *Bombyx mori*, the first double bond is introduced at C16:CoA fatty acid by Z11 desaturase to form Z11-16:CoA (Matsumoto et al., 2007). The desaturation reaction is common in *Heliothis subflexa, Spodoptera litura*, and *Antheraea pernyi* (Choi et al., 2002; Lin et al., 2018). In *H. subflexa*, desaturation of C18:CoA is catalyzed by Z11 desaturase to produce Z11-C18:CoA (Choi et al., 2002). Both Z11 desaturase (converts C16:CoA to Z11-C16:CoA) and E11 desaturase (converts C14:CoA to E11-C14:CoA) have been identified in *S. litura* (Lin et al., 2018). Several other desaturases that catalyze double bond generation in saturated fatty acyl have been identified. For instance, in *Streltzoviella insularis*, Δ5 desaturases (Z5 and E5) catalyze the conversion of C16:CoA to (E/Z)5-C16:CoA, and Z9 desaturases catalyze the conversion of C16:CoA to Z9-C16:CoA (Yang et al., 2020). In *A. pernyi*, E6 desaturase catalyzes C16:CoA to E6-C16:CoA (Wang et al., 2010a). In *Grapholita molesta* and *Grapholita dimorpha*, Δ10 desaturase (Z10 and E10) desaturates C14:CoA to form (E/Z)10-C14:CoA (Jung and Kim, 2014). Several desaturases exhibit the specificity for mono-unsaturated substrates. In *S. litura*, Z9 desaturase introduces a second double bond in E11-C14:CoA to form Z9, E11-C14:CoA, while E12 desaturase converts Z9-C14:CoA to Z9, E12-C14:CoA (Lin et al., 2018). In *A. pernyi*, E6 desaturase converts C16:CoA to E6-C16:CoA, and Z11 desaturase converts E6-C16:CoA to E6, Z11-C16:CoA (Wang et al., 2010a). Likewise, E5, Z7-C12:CoA is produced by the desaturation of Z7-C12:CoA by E5 desaturase in *Thysanoplusia intermixta* (Ono et al., 2002). Many moth and other Lepidopteran pheromone intermediates can be desaturated by applying different combinations, temporal orders, and stereospecificity of diverse desaturases.

## Fatty Acid Reductases

Fatty acid reductases catalyze the reduction of fatty-acyl pheromone precursors to their corresponding alcohols. In moth, many types of FARs have been identified and their substrate specificity has been found to produce species-specific pheromones (Figure 1). Some FARs exhibit broad substrate preferences. For example, the first pheromone-gland fatty acyl reductase (pgFAR) isolated from the *B. mori* shows its activity on a broad range of saturated and monounsaturated C14- and C18-acyl precursors (Moto et al., 2003). In three species of *Yponomeuta*, a single pgFAR has been found to efficiently reduce saturated and unsaturated C14- and C16-acyl precursors (Liénard et al., 2010). The pgFARs have been found to possess a general selectivity for C8-C16 fatty acyl precursors in four closely related heliothine moths (Hagström et al., 2012). Some FARs also reduce specific pheromone intermediates. In two sex pheromone races of *Ostrinia nubilalis*, the allelic variation in the gene coding for pgFAR has been observed to cause distinct substrate stereoselectivity (E11-C14:acid and Z11-C14:acid), which contributes to the intraspecific reproductive isolation in moths (Lassance et al., 2010). In *Spodoptera exigua*, a highly selective pgFAR I has been reported, which catalyzes the reduction of Z11-C16:acyl to produce moth pheromone signals (Antony et al., 2016). The substrate selectivity of *pg*FARs plays an essential role in generating species-specific signals.

## The Modification Enzymes

Fatty acid reductases catalyze the conversion of the functional groups of fatty-acyl pheromone precursors to the hydroxyl group. Likewise, aldehyde-producing oxidases, acetyltransferases, and other enzymes can also modify the pheromones in many moths (Figure 1). Some oxidases transform fatty alcohols into aldehydes. The aldehydic sex pheromone components in *Heliothis zea* are catalyzed by cuticular alcohol oxidases, and the oxidases are also responsible for the conversion of the primary fatty alcohols to fatty aldehydes (Dou et al., 2020). In *H. subflexa* and *H. virescens*, the major pheromone component is Z11-hexadecenal (Z11-C16:Ald), which is produced by the oxidation of the precursor Z11-hexadecenol (Z11-C16:OH) (Choi et al., 2002). The sex pheromone component of *Amyelois transitella*, Z11, Z13-C16:Ald, is probably produced from Z11, Z13-C16:OH in the PG by oxidation (Wang et al., 2010b). In some moth species of *Choristoneura fumiferana* (Roscoe et al., 2016), *S. litura* (Lin et al., 2018), and *S. insularis* (Yang et al., 2020), the acetyl transferases catalyze the conversion of fatty alcohols to acetate ester pheromones.

Thus, diverse Type-I pheromones are generated by the co-catalyzation of differing combinations, temporal orders, and substrate specificities of desaturases, reductases, oxidases, and modification enzymes (Matsumoto, 2010).

## Metabolic Engineering Strategies for Fatty Acid Production in *Saccharomyces cerevisiae*

As a model species, *S. cerevisiae* has been used to produce a variety of different fatty acids or fatty acid-derived products (Wei et al., 2019; Gao et al., 2020; Guan et al., 2020). Most Lepidopteran sex pheromones are fatty acid alcohols, aldehydes, or fatty alcohol acetates with chain lengths of 10-18 carbons. *S. cerevisiae* cells mainly produce C16 and C18 fatty acids (Tehlivets et al., 2007; Wei et al., 2017b), which are suitable as potential chassis for the microbial synthesis of insect sex pheromones (Supplementary Figure 1).

Acetyl-CoA is the precursor for fatty acid biosynthesis. In mitochondria, acetyl-CoA mainly enters the TCA cycle to be oxidized and release energy. In the cytoplasm, pyruvate is converted to acetyl-CoA under the catalysis of pyruvate decarboxylase, acetaldehyde dehydrogenase (ALD), and two acetyl-CoA synthetases (ACS1 and ACS2) (Krivoruchko et al., 2015; Zhang et al., 2018; Wang M. et al., 2020). The carboxyl group is transferred to acetyl-CoA to yield malonyl-CoA catalyzed by acetyl-CoA carboxylase (ACC1). Then, acetyl-CoA and malonyl-CoA are catalyzed by FAS for fatty acid synthesis and elongation (Tehlivets et al., 2007). In *S. cerevisiae*, cytosolic FAS is capable of synthesizing fatty acids up to C20 *in vitro*. The elongation enzymes (ELO) can increase the length of the fatty acids up to C26 in yeasts (Rössler et al., 2003). Normally, ELO1 extends C12-C16 fatty acyl-CoAs to C16-C18 fatty acids, ELO2 extends palmitoyl-CoA and stearoyl-CoA up to C22 fatty acids, and ELO3 catalyzes the synthesis of C20-C26 fatty acids from C18-CoA (Oh et al., 1997; Rössler et al., 2003; Wenning et al., 2017). Approximately 70-80% of yeast fatty acids are monounsaturated, and the desaturation reactions are catalyzed by OLE1, the endoplasmic reticulum membrane-bound Δ9 fatty acid desaturase (Martin et al., 2007). Metabolic engineering strategies for the biosynthesis of fatty acids mainly include increasing the content of fatty acid precursors and cofactors, eliminating the competing pathways, and regulating the activity of fatty acid synthases and elongation enzymes (Supplementary Figure 1).

Cytosolic acetyl-CoA is the primary substrate for *de novo* FAS biosynthesis in yeast. Deletion of alcohol dehydrogenase (ADH) genes could reduce ethanol formation from acetaldehyde, and the overexpression of a heterologous mutant acetyl-CoA synthase from *Salmonella enterica* (SeAcs^*L*641*P*^) combined with the overexpression of *ALD* (more specifically *ALD6*) could increase the flux toward acetyl-CoA-derived products (Shiba et al., 2007). Alternatively, engineering the cytosolic pyruvate dehydrogenase (PDH) bypass pathway (Kozak et al., 2014), ATP-citrate lyase (ACL) pathway, and the phosphoketolase pathway could further enhance acetyl-CoA biosynthesis (de Jong et al., 2014). Expression of an ATP-independent PDH complex from *Enterococcus faecalis* and ACL could increase the pool of cytosolic acetyl-CoA in *S. cerevisiae* (Supplementary Figure 1; Kozak et al., 2014; Feng et al., 2015).

Malonyl-CoA is the rate-limiting compound in *de novo* fatty acid biosynthesis in yeast. Overexpression of *ACC1* gene in *S. cerevisiae* was found to increase the levels of fatty acids (Shin et al., 2012). Plasmid-based overexpression of endogenous *ACC1* increased the total fatty acid production from 42.7 mg/L to 63.2 mg/L (∼1.48-fold) (Runguphan and Keasling, 2014). The activity of ACC1 is negatively regulated by snf1-dependent phosphorylation, and the introduction of mutations in two phosphorylation sites of *ACC1* (*ACC1*^*S*659*A, S*1157*A*^) increased ACC1 activity and total fatty acid content (Shi et al., 2014). When the third mutation was introduced in *ACC1*^*S*659*A, S*1157*A*^, the resulting strain with *ACC1*^*S*686*A, S*659*A, S*1157*A*^ could produce a higher amount malonyl-CoA, and the titer of 3-hydroxypropionic acid — a malonyl-CoA-derived compound — improved by 1.5-fold (Chen et al., 2018).

The cofactor NADPH is required in the cell for *de novo* fatty acid synthesis in yeast. A recent metabolic flux analysis indicated that 60% of all NADPH is consumed for glutamate biosynthesis by the NADP^+^-dependent glutamate dehydrogenases GDH1 and GDH3 (d’Espaux et al., 2017). Deletion of the *GDH1* gene in yeast resulted in a 2.7-fold improvement in fatty alcohol production (d’Espaux et al., 2017). Xylulose-5-phosphate, which is produced by the pentose phosphate (PP) pathway, acts as the precursor of the phosphoketolase (PHK) pathway. The combination of PHK and PP pathways in *S. cerevisiae* led to increased cytosolic NADPH levels and subsequently improved production of fatty acid ethyl esters (de Jong et al., 2014). Downregulation of *PGI1* (encoding glucose-6-phosphate isomerase 1) and enhancing PP pathway flux by overexpression of *ZWF1* (encoding glucose-6-phosphate dehydrogenase), *GND1* (encoding 6-phosphogluconate dehydrogenase, decarboxylating 1), *TKL1* (encoding transketolase-1), and *TAL1* (encoding transaldolase) could increase NADPH supply and significantly improve the production of free fatty acids (FFAs) (Yu et al., 2018).

Another effective way to increase fatty acids production involves eliminating the competing metabolic pathways, such as β-oxidation and triacylglycerol production (Wei et al., 2017a, 2018). The β-oxidation pathway is often stalled by deletion of *FAA* (encoding acyl-CoA synthetase), *PXA* (encoding the peroxisomal long-chain acyl-CoA transporter complex), and/or *POX1* (encoding fatty acyl-CoA oxidase, catalyzing the first step of β-oxidation). Disruption of β-oxidation by knockouts in *FAA2*, *PXA1*, and *POX1* genes increased intracellular fatty acid levels by 55% compared to that in the control strain BY4741 (Leber et al., 2015). The disruption of *FAA1* in *S. cerevisiae* B-1 strain resulted in a two-fold increase in fatty acid secretion level (Michinaka et al., 2003), while a double deletion of *FAA1* and *FAA4* in yeast significantly increased the production of FFAs (Scharnewski et al., 2008; Li et al., 2014; Runguphan and Keasling, 2014; Zhou et al., 2016). Simultaneous deletion of *FAA1*, *FAA4*, and *POX1* further increased the production of fatty acids by 31% compared to strain with only *FAA1* and *FAA4* deletion (Li et al., 2014). A *S. cerevisiae* strain with multi-gene deletions (*faa1*Δfaa*4*Δ*fat1*Δ*faa2*Δ*pxa1*Δ*pox1*Δ) was able to produce 1.3 g/L extracellular FFAs, which is higher than 490 mg/L FFAs production in a strain with triple deletions of *faa1*Δ*faa4*Δ*fat1*Δ (Leber et al., 2015).

Overexpression of the FAS complex by replacing the native *FAS1* and *FAS2* promoters with the strong constitutive P*_*TEF*_*_1_ promoter could increase total fatty acid production as well as lipid content (Runguphan and Keasling, 2014). In fact, plasmid-based overexpression of the *E. coli* acyl-ACP thioesterase (‘*TesA*) in *S. cerevisiae* led to the production of 5 mg/L of FFAs, eight-times that produced by the background strain (0.6 mg/L) (Runguphan and Keasling, 2014). Overexpression of ‘*TesA* in *S. cerevisiae* WRY1 strain (all fatty acid biosynthesis genes under P_*TEF*1_ promoter) improved FFAs production levels to 52 mg/L (Runguphan and Keasling, 2014). Likewise, expressing FAS from *Rhodospuridium toruloides* (RtFAS) in yeast increased the total lipid as well as FFA content (Zhou et al., 2016). In another report, a combination strategy, involving blockage of fatty acid activation and degradation, introducing an optimized acetyl-CoA pathway, expressing a more efficient FAS, and overexpressing the endogenous acetyl-CoA carboxylase increased FFA titer to 10.4 g/L (Zhou et al., 2016).

The length and saturation of fatty acids in *S. cerevisiae* can be modulated. When a thioesterase from *Acinetobacter baylyi* (’*Ac*TesA) was embedded into reaction compartments of fungal FASs, it led to 5-13 times more production of extracellular short/medium-chain fatty acids compared to that in the wild-type strains (Zhu et al., 2017). In one study, *S. cerevisiae* was successfully engineered to produce very-long-chain fatty acids (C16-C18 and C22-C24 VLCFAs) and derived chemicals by incorporating a heterologous FAS I system from *Mycobacterium vaccae*. By the introduction of endogenous yeast fatty acid elongation system, C22-C26 fatty acids could be selectively produced (Yu et al., 2017). By tailoring the bacterial carboxylic acid reductase from *Mycobacterium marinum* (MmCAR) via directed evolution and rational design, introduction of the MmCAR variants and metabolic engineering strategies successfully led to the establishment of a *S. cerevisiae* platform with the capability of medium-chain fatty alcohol production (Hu et al., 2020). Furthermore, modulation of *ACC1* and *ELO1* expression led to increased titers of C18:0 and C18:1 (Bergenholm et al., 2018).

## Production of Insect Sex Pheromones in Yeasts

Compared with conventional insecticides, insect sex pheromones are specific for pest management and are thus environmentally friendly. Some insect sex pheromone biosynthetic enzymes and pathways have been identified and functionally analyzed, providing the possibility to produce pheromones by synthetic biology-based strategies. A pheromone-gland-specific *FAR* gene (encoding FAR) of the silkmoth was expressed in *S. cerevisiae*, and the resulting yeast strain could produce E10, Z12-C16:OH, which induced typical mating behavior in male *B. mori* (Moto et al., 2003). Co-expression of a Δ11 fatty acyl-CoA desaturase gene and a reductase gene of *Agrotis segetum* in *S. cerevisiae* led to the production of a set of long-chain fatty acids and alcohols that do not occur naturally in yeast, and the titer of Z11-C16:OH was 19.5 μg/L. Moreover, the oxidized extracts from the yeast cells were found to induce specific electrophysiological activity in male antennae of *H. virescens* (Hagström et al., 2013b).

Insect pheromones of Z11-C16:OH and Z9-tetradecenol (Z9-C14:OH) have been produced by engineered oleaginous yeast, *Yarrowia lipolytica.* The combined activity of a desaturase from *A. transitella* (AtrΔ11) and a reductase from *Helicoverpa armigera* (HarFAR) resulted in the production of 1.7 mg/L Z11-C16:OH (Table 1; Holkenbrink et al., 2020). Likewise, the combination expression of a desaturase from *Drosophila melanogaster* (DmeΔ9), reductase HarFAR, and acetyltransferase ATF1 of *S. cerevisiae* led to the production of 7.3 mg/L Z9-C14:OAc, which is the main sex pheromone component of the fall armyworm *Spodoptera frugiperda*. (Table 1; Holkenbrink et al., 2020). Several strategies, including preventing endogenous fatty alcohol degradation, inhibiting acyl-CoA degradation, reducing the flux toward storage lipids, and increasing the supply of tetradecanoyl-CoA precursor, have been applied to improve pheromones production; consequently, the engineered *Y. lipolytica* strains could produce 73.6 mg/L of Z9-C14:OH (15-fold increase in titer over the background strain) and 2.57 g/L of Z11-C16:OH (Table 1; Holkenbrink et al., 2020). Introduction of a point mutation into the α-subunit of FAS (*FAS2*^*I*1220*F*^), and overexpression of an optimal combination of a fatty acyl-CoA desaturase (FAD; Lbo_PPTQ) from *Lobesia botrana*, FAR (HarFAR) from *H. armigera* and the gene encoding native FAS1 led to a final Z11-14:OH titer of 188.1 mg/L in fed-batch fermentation (Table 1; Petkevicius et al., 2021). In another study, expression of the gene encoding FAR, *BlapFAR*4 from *Bombus lapidarius* or *BlucFAR1* from *Bombus lucorum* in *Y. lipolytica*, led to the production of bumblebee pheromones consisting of long-chain fatty alcohols. The titer of saturated fatty alcohols with C18-C24 was 166.6 mg/L, while the titer of C16 FA-OHs (C16:0-OH and C16:1-OH) was 14.6 mg/L (Table 1; Hambalko et al., 2020). However, in *S. cerevisiae*, expression of *BlucFAR1* produced only a small amount of FA-OHs (6.9 mg/L), while the expression of *BlapFAR4* led to the production of 79 mg/L of C16-OHs (Table 1; Tupec et al., 2019). The difference in titers and chain length of FA-OH products in engineered *Y. lipolytica* and *S. cerevisiae* may be due to the variations in substrate availability, hinting at the selection of different yeast chassis cells for the biosynthesis of different sex pheromones based on yeast fatty acid profiles (Wei et al., 2017b). As *Y. lipolytica* is an oleaginous yeast and robust to diverse substrates, *Y. lipolytica* has more potential to be an ideal insect pheromone production cell factory (Dobrowolski et al., 2019; Vasconcelos et al., 2019).

**TABLE 1:** A summary of insect pheromones produced by various engineered yeasts.

Insect sex pheromones	Derived Insect species	Yeast species	Yeast products	Fermentation scale	Titers of product	References
E10, Z12-C16:OH	*B. mori*	*S. cerevisiae*	E10,Z12-C16:OH	Flask	ND^*a*^	Moto et al., 2003
Z11-C16:Ald	*H. virescens*	*S. cerevisiae*	Z11-C16:OH	Flask	19.5 μg/L	Hagström et al., 2013b
Z11-C16:Ald	*H. armigera*	*Y. lipolytica*	Z11-C16:OH	Bioreactor	2.57 g/L	Holkenbrink et al., 2020
Z9-C14:OAc	*S. frugiperda*	*Y. lipolytica*	Z9-C14:OH	Flask	73.6 mg/L	Holkenbrink et al., 2020
*Z*11-C14:OAc	*O. nubilalis*	*Y. lipolytica*	*Z*11-C14:OH	Bioreactor	188.1 mg/L	Petkevicius et al., 2021
C16-C18 fatty alcohols	*B. lapidarius*	*Y. lipolytica*	C16 FA-OHs	Flask	14.6 mg/L	Hambalko et al., 2020
C16-C18 fatty alcohols	*B. lucorum*	*Y. lipolytica*	C18-C24 FA-OHs	Flask	166.6 mg/L	Hambalko et al., 2020
C16-C18 fatty alcohols	*B. lapidarius*	*S. cerevisiae*	C16 FA-OHs	Flask	79 mg/L	Tupec et al., 2019
C16-C18 fatty alcohols	*B. lucorum*	*S. cerevisiae*	C18-C26 FA-OHs	Flask	6.9 mg/L	Tupec et al., 2019

*^*a*^Data not provided.*

As cell factories, *S. cerevisiae* and other yeasts mainly produce C16 and C18 fatty acids (Wei et al., 2017b; Wang J. et al., 2020), that are suitable for the biosynthesis of C16 and C18 pheromones. Currently, various metabolic engineering strategies, such as increasing substrate precursors and cofactors, eliminating competing pathways, and modulation of FAS and other keystone enzymes in sex pheromone biosynthetic pathways, have been developed to improve the production of fatty acids and their derivatives in yeasts. Engineered yeasts can produce a high level of Lepidopteran and a few other insect sex pheromones. Strategies to increase pheromone production using synthetic biology approaches require designing optimal pheromone biosynthetic pathway, and protein engineering of key enzymes with high activities. It is also necessary to identify more suitable keystone genes for insect pheromone production based on omics technologies.

## Conclusion and Future Perspective

The cost of chemically synthesized pheromones is high, therefore, mating disruption applications currently primarily target higher-value crops (Ioriatti and Lucchi, 2016). Biosynthesis of insect sex pheromone using engineered microbial strains is one of the most promising environmentally friendly strategies for large-scale commercial production of pheromones at a relatively low cost. An increasing number of FADs and FARs and other functional modification enzymes involved in the biosynthesis of Lepidopteran sex pheromones have been successfully identified and functionally characterized. However, characterizing more efficient insect sex pheromone enzymes and adaption of insect sex pheromone pathways to yeast cell factories still need further studies.

As cell factories, *S. cerevisiae* and other yeasts produce fatty acids that are appropriate for C16 and C18 pheromone biosynthesis. Various metabolic engineering strategies, such as increasing levels of the biosynthetic precursors and cofactor, eliminating competing pathways, and regulating the activity of FAS and elongation enzymes, have been developed in yeast to increase the production of fatty acids and to modify chain length and saturation. With such synthetic biology strategies, engineering of *S. cerevisiae*, *Y. lipolytica* or other yeasts to produce Lepidopteran sex pheromones has achieved considerable success. With further development and application of advanced yeast tools, such as high throughput screening strategy (Tan et al., 2020), metabolic mass transfer strategy (Xue et al., 2021), and efficient gene editing tools for non-conventional oleaginous yeasts (Shan et al., 2021), large-scale commercial production of sex pheromones in engineered yeasts with a high titer, rate and yield would be possible, and will also facilitate moth-mating disruption using biosynthetic sex pheromones in a cost-efficient manner. In the future, the application of yeast-based sex pheromones will lead to eco-friendly agriculture with a green pest control strategy.

## Author Contributions

YW conceived the study. XZ and YW drafted the manuscript and designed the whole study. XZ, QM, and YW prepared the figures. BJ, XX, and LQ revised and polished the manuscript. All authors read, revised, and approved the manuscript.

## Conflict of Interest

The authors declare that the research was conducted in the absence of any commercial or financial relationships that could be construed as a potential conflict of interest.

## Publisher’s Note

All claims expressed in this article are solely those of the authors and do not necessarily represent those of their affiliated organizations, or those of the publisher, the editors and the reviewers. Any product that may be evaluated in this article, or claim that may be made by its manufacturer, is not guaranteed or endorsed by the publisher.
