# Supplementary material for: Developments in Fatty Acid-Derived Insect Pheromone Production Using Engineered Yeasts
Source: Front Microbiol. 2021 Nov 11;12:759975. doi: 10.3389/fmicb.2021.759975 (PMC8632438; doi:10.3389/fmicb.2021.759975)
Supplement: Supplementary file 1 [file Presentation_1.pdf]

Developments in insect pheromone production using engineered yeasts

Xiaoling Zhang[1,2], Qin Miao[1,2], Xia Xu[1], Boyang Ji[3], Lingbo Qu[2,4], Yongjun Wei[1,2*]

[1]Key Laboratory of Advanced Drug Preparation Technologies, Ministry of Education, School of Pharmaceutical Sciences, Zhengzhou University, Zhengzhou 450051, PR China
[2]Laboratory of Synthetic Biology, Zhengzhou University, Zhengzhou 450051, PR China
[3]Department of Biology and Biological Engineering, Chalmers University of Technology, Gothenburg, Sweden
[4]College of Chemistry, Zhengzhou University, Zhengzhou 450001, Henan, China

[*]Corresponding author
Yongjun Wei
E-mail: yongjunwei@zzu.edu.cn

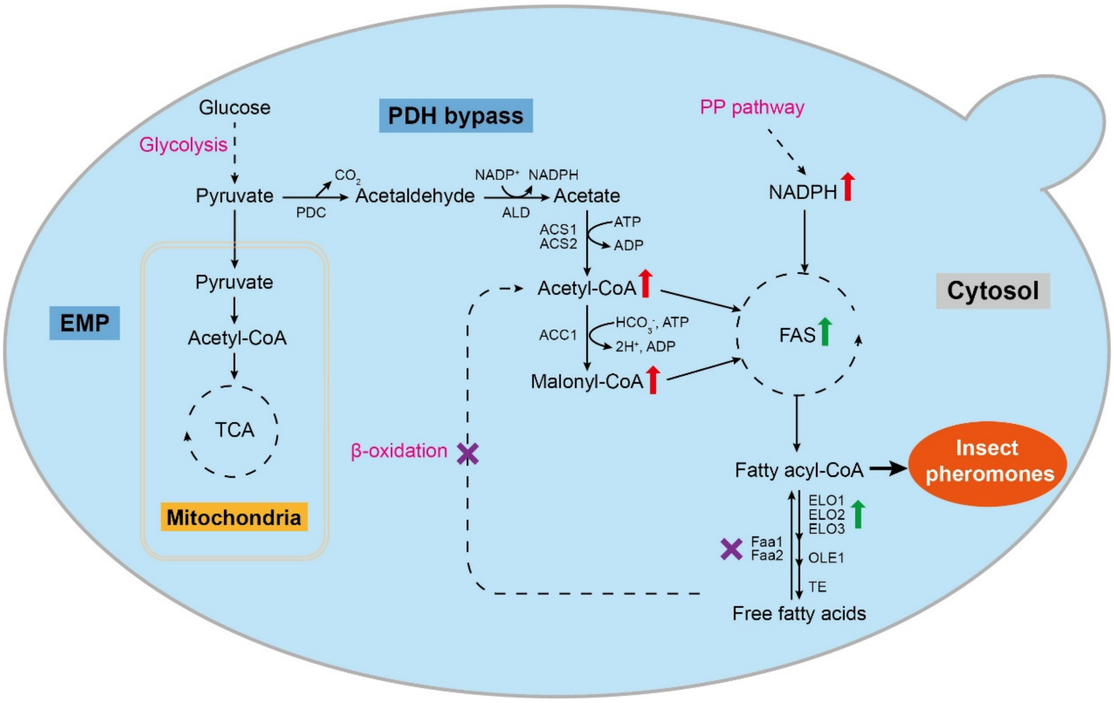

Supplementary Figure 1 An overview of the fatty acid metabolic pathway in *Saccharomyces cerevisiae*. Arrows in red represent the strategy to increase the content of biosynthetic precursors and cofactors in *S. cerevisiae* for fatty acid production. Arrows in green represent modulation of fatty acid synthase and elongation enzymes to obtain desired fatty acids. Purple cross symbols represent eliminating competing pathways for fatty acid production. Solid arrows indicate single enzymatic steps, and dashed arrows indicate multiple enzymatic steps. EMP, Embden-Meyerhof-Parnas pathway; PDH bypass, pyruvate dehydrogenase bypass pathway; TCA, tricarboxylic acid cycle; FAS, fatty acid synthase; PP pathway, pentose phosphate pathway; PDC, pyruvate decarboxylase; ALD, acetaldehyde dehydrogenase; ACS, acetyl-CoA synthetase; ACC, cytosolic acetyl-CoA carboxylase; FAA, fatty acyl-CoA synthetase; ELO, elongation enzymes; OLE, fatty acid desaturase; TE, thioesterase.
